# Supplementary material for: Hypertension care cascade in the Ingwavuma rural community, uMkhanyakude District, KwaZulu-Natal province of South Africa
Source: PeerJ. 2021 Nov 11;9:e12372. doi: 10.7717/peerj.12372 (PMC8590801; doi:10.7717/peerj.12372)
Supplement: Supplemental Information 1 [file peerj-09-12372-s001.pdf]

STUDY TITLE: PREVALENCE, AWARENESS, HEALTHCARE UTILIZATION AND BURDEN OF CARDIOVASCULAR DISEASE AND ITS RISK FACTORS IN INGWAVUMA, KWAZULU-NATAL

Interviewer's name ……………………………………………………………………………….

Interview date (device date)………………..

Interview starting time (device time)…………….

Introduction: My name is …… I am part of the research team and we are interviewing people in the various areas of Ingwavuma for the following: to understand the community's perception on overweight and obesity, hypertension, type 2 diabetes mellitus and cardiovascular disease, to determine prevalence of overweight and obesity, hypertension and type 2 diabetes mellitus, to understand the utilization of healthcare services by patients with hypertension and type 2 diabetes mellitus and to determine the burden of cardiovascular disease.

Confidentiality and consent: "I am going to ask you some personal questions that some people find difficult to answer. Your answers are completely confidential. You do not have to answer any questions that you do not want to answer and you may end this interview anytime you want to. However, your honest answers will help us to understand what people of Ingwavuma know about cardiovascular disease and its risk factors. Your participation will also help us to understand the prevalence of cardiovascular disease risk factors, use of healthcare services in the Ingwavuma area and the burden of these diseases. We would greatly appreciate your help in responding to this survey. It will take about 30 – 45 minutes to ask the questions and record measurements. Would you be willing to participate?"

Yes	☐
No	☐

(Signature of respondent on KoBoCollect certifying informed consent)

…………………………………………………………………………………………………

SECTION A: INTERVIEW DETAILS AND BACKGROUND CHARACTERISTICS

No	Questions	Responses and coding categories
A1	Name of interviewee	
A2	Gender of interviewee	
A3	Date of birth ("can I see your identity document")	
A4	Age of interviewee (*for those without identity documents), ask for health card and search for age or settle for self-reported age*)	
A5	Name of area	
A6	Name of village	
A7	Ward	
A8	House number (check household number from malaria control card)	
A9	Mobile number	
A10	Marital status	Single (never married) 0<br>Married 1<br>Cohabiting 2<br>Divorced 3<br>Widowed 4
A11	Have you ever attended school?	No 0<br>Yes 1
A12	What is the highest level of school you completed:<br><br>**select one**	Primary 0<br>Secondary 1<br>College level 2<br>Above college level 3
A13	Occupational status	Student 0<br>Self-employed 1<br>Employed by government 2<br>Private company 3<br>Retired 4<br>Unemployed 5
A14	How many other people are employed in your household?	***Enter the number***
A15	Do you receive any social grant?	No 0<br>Yes 1
A16	Beside you, how many other members in the household receive social grants?	***Enter the number***
A17	How long have you lived in this area?	Number of years |__|__| Number of months |__|__|

B. GENERAL HEALTH QUESTIONS

No	Questions	Responses and coding categories	
B1	Where do you generally get healthcare services?	Public clinic/hospital<br>Private clinic/hospital<br>Private pharmacy<br>Traditional healer<br>Faith healer	0<br>1<br>2<br>3<br>4
B2	Do you sometime fail to seek care when ill?	No<br>Yes	0<br>1
B3	List the reasons that make you not to seek care when ill	***List the factors***	
B4	What are the most dominant health conditions diseases in this area?	***List the conditions/diseases***	
B5	Which health conditions or diseases contribute to the most deaths in this area?	***List the conditions/diseases***	
B6	Have you ever heard of cardiovascular disease?	No<br>Yes	0<br>1
B7	Do you know cardiovascular disease risk factors?	No<br>Yes<br>**If No skip to …**	0<br>1
B8	Can you list the cardiovascular disease risk factors	***List the factors***	
B9	Are there ways to reduce the risk of cardiovascular disease?	No<br>Yes	0<br>1
B10	Can you list the possible ways of reducing the risk of cardiovascular disease?	***List the ways***	
B11	Which age group is at most risk of developing cardiovascular disease?	18 – 30 years<br>30 – 40 years<br>40 – 50 years<br>Above 50 years<br>All age groups	0<br>1<br>2<br>3<br>4

C. TOBACCO

Now I am going to ask you some questions about tobacco use.			
**Serial**	**Questions**	**Responses and coding categories**	
C1	Do you currently smoke any tobacco products, such as cigarettes, cigars or pipes?	No<br>Yes<br>**If No skip to C8**	0<br>1
C2	Do you smoke daily?	No<br>Yes	0<br>1
C3	How old were you when you first started smoking?	Enter age in years<br>Don't know	--
C4	On average, how many of the following products do you smoke each day?	**Enter number of:**<br>Manufactured cigarettes<br>Hand-rolled cigarettes<br>Pipes full of tobacco<br>Other	 --<br>--<br>--<br>--
C5	During the past 12 months, have you tried to stop smoking?	No<br>Yes	0<br>1
C6	Did you visit a doctor or health worker during the past 12 months?	No<br>Yes	0<br>1
C7	During any visit, were you advised to quit smoking tobacco?	No<br>Yes	0<br>1
C8	In the past, did you ever smoke any tobacco products?	No<br>Yes<br>**If No skip to C12**	0<br>1
C9	In the past, did you ever smoke daily?	No<br>Yes	0<br>1
C10	How old were you when you stopped smoking?	Enter age in years<br>Don't know	--
C11	How long ago did you stop smoking tobacco?	Years<br>Months<br>Weeks<br>Don't know	--<br>--<br>--
C12	Do you currently use any smokeless tobacco products such as snuff, chewing tobacco?	No<br>Yes	0<br>1
C13	Do you think smoking tobacco can cause illness or increase chances of developing chronic non-communicable conditions?	No<br>Yes<br>**If No skip to D1**	0<br>1
C14	What illnesses or chronic conditions are likely to result from tobacco smoking	Enter list of diseases	

D. ALCOHOL CONSUMPTION

The next questions ask about the consumption of alcohol.			
**Serial**	**Questions**	**Response and coding categories**	
D1	Have you ever consumed any alcohol such as beer, wine or spirits?	No<br>Yes<br>**If No skip to E1**	0<br>1
D2	What type of alcoholic drinks did you consume?	Beer<br>Wine<br>Spirits<br>Traditional brew<br>Other	0<br>1<br>2<br>3<br>4
D3	Have you consumed any alcohol within the past 12 months?	No<br>Yes<br>**If No skip to E1**	0<br>1
D4	Have you stopped drinking due to health reasons, such as a negative impact on your health or on the advice of a doctor or other health worker?	No<br>Yes	0<br>1
D5	During the past 12 months, how frequently have you had at least one standard alcoholic drink?	Daily<br>5-6 days per week<br>3-4 days per week<br>1-2 days per week<br>1-3 days per month	0<br>1<br>2<br>3<br>4
D6	Have you consumed any alcohol within the past 30 days?	No<br>Yes<br>**If No skip to D12**	0<br>1
D7	During the past 30 days, on how many occasions did you have at least one standard alcoholic drink?	Enter number<br>Don't know	--
D8	During the past 30 days, when you drank alcohol, how many standard drinks on average did you have during one drinking occasion?	Enter number<br>Don't know	--
D9	During the past 30 days, what was the largest number of standard drinks you had on a single occasion, counting all types of alcoholic drinks together?	Enter number<br>Don't know	--
D10	During the past 30 days, how many times did you have six or more standard drinks in a single drinking occasion?	Enter number of times<br>Don't know	--

D11	During each of the past 7 days, how many standard drinks did you have each day?	Enter number for each day<br>Monday<br>Tuesday<br>Wednesday<br>Thursday<br>Friday<br>Saturday<br>Sunday
D12	During the past 12 months, how often have you found that you were not able to stop drinking once you had started?	Never 0<br>Sometime 1<br>Often 2
D13	During the past 12 months, how often have you needed a first drink in the morning to get yourself going after a heavy drinking session?	Never 0<br>Daily or almost daily 1<br>Weekly 2<br>Monthly<br>3<br>Less than monthly 4
D14	Did you ever stop drinking alcohol?	No 0<br>Yes 1<br>**If No skip to E1**
D15	What made you stop drinking alcohol?	List the reasons ………………......

E. DIET

The next questions ask about the fruits and vegetables that you usually eat		
**Serial**	**Questions**	**Response and coding categories**
E1	Do you eat fruits?	No 0<br>Yes 1<br>**If No skip to E4**
E2	In a typical week, on how many days do you eat fruits?	Enter number of days --<br>Don't Know
E3	How many servings of fruit do you eat on one of those days?	Enter number of servings --<br>Don't Know
E4	Do you eat vegetables?	No 0<br>Yes 1<br>**If No skip to F1**
E5	In a typical week, on how many days do you eat vegetables?	Enter number of days --<br>Don't Know
E6	What quantity of vegetables do you eat on one of those days?	Little 0<br>Moderate 1<br>Quite a lot 2

F. DIETARY SALT

With the next questions, we would like to learn more about salt in your diet. Dietary salt includes ordinary table salt, unrefined salt such as sea salt, iodized salt, salty stock cubes and powders, and salty sauces such as soy sauce or fish sauce. The following questions are on adding salt to the food right before you eat it, on how food is prepared in your home, on eating processed foods that are high in salt such as fried chips and questions on controlling your salt intake. Please answer the questions even if you consider yourself to eat a diet low in salt.			
**Serial**	**Questions**	**Response and coding categories**	
F1	How often do you add salt or a salty sauce such as soy sauce to your food right before you eat it or as you are eating it?	Never<br>Sometimes<br>Always	0<br>1<br>2
F2	How often is salt, salty seasoning like Norox or Aromat, or a salty sauce added in cooking or preparing foods in your household?	Never<br>Sometimes<br>Always	0<br>1<br>2
F3	How often do you eat processed food high in salt? By processed food high in salt, I mean foods that have been altered from their natural state, such as packaged salty snacks, canned salty food including pickles and preserves, salty food prepared at a fast food restaurant, cheese, bacon and processed meat.	Never<br>Sometimes<br>Always	0<br>1<br>2
F4	How much salt or salty sauce do you think you consume?	Far too little<br>Little<br>Just the right amount<br>Too much<br>Far too much	0<br>1<br>2<br>3<br>4
F5	How important to you is lowering the salt in your diet?	Not at all important<br>Somewhat important<br>Very important<br>Don't know	0<br>1<br>2<br>3
F6	Do you think that too much salt or salty sauce in your diet could cause a health problem?	No<br>Don't know<br>Yes<br>**If No or Don't know Skip to**	0<br>1<br>2

F7	If yes, can you list the health problems that may be caused by consuming too much salt or salty sauce	List…………………………
**F. DIETARY SALT Continued**<br>**Do you do any of the following on a regular basis to control your salt intake?**		
F8	Limit consumption of processed foods	No 0<br>Yes 1
F9	Look at the salt or sodium content on food labels	No 0<br>Yes 1
F10	Buy low salt/sodium alternatives	No 0<br>Yes 1
F11	Use spices other than salt when cooking	No 0<br>Yes 1
F12	Avoid eating foods prepared outside of a home	No 0<br>Yes 1
F13	Do other things specifically to control your salt intake	No 0<br>Yes 1
F14	Other (please specify)	List the measures

G. PHYSICAL ACTIVITY

Next I am going to ask you about the time you spend doing different types of physical activity in a typical week. Please answer these questions even if you do not consider yourself to be a physically active person.<br>Think first about the time you spend doing work. Think of work as the things that you have to do such as paid or unpaid work, household chores, harvesting food/crops, fishing or hunting for food, seeking employment. In answering the following questions 'vigorous-intensity activities' are activities that require hard physical effort and cause large increases in breathing or heart rate, 'moderate-intensity activities' are activities that require moderate physical effort and cause small increases in breathing or heart rate.		
**Serial**	**Questions**	**Response and coding categories**
G1	Does your work involve vigorous-intensity activity that causes large increases in breathing or heart rate like [carrying or lifting heavy loads, digging or construction work] for at least 10 minutes continuously?	No 0<br>Yes 1<br><br>**If No skip to G5**
G2	In a typical week, on how many days do you do vigorous-intensity activities as part of your work?	Enter number of days --
G3	How much time do you spend doing vigorous-intensity activities on a typical day?	Hours --<br>Minutes --

G4	Does your work involve moderate-intensity activity, that causes small increases in breathing or heart rate such as brisk walking [or carrying light loads] for at least 10 minutes continuously?	No 0<br>Yes 1
G5	In a typical week, on how many days do you do moderate-intensity activities as part of your work?	Enter number of days
G6	How much time do you spend doing moderate-intensity activities at work on a typical day?	Hours --<br>Minutes --
**Travel to and from places**<br>The next questions exclude the physical activities at work that you have already mentioned. Now I would like to ask you about the usual way you travel to and from places. For example travelling to work, for shopping or place of worship.		
G7	Do you walk or use a bicycle for at least 10 minutes continuously to get to and from places?	No 0<br>Yes 1
G8	In a typical week, on how many days do you walk or bycycle for at least 10 minutes continuously to get to and from places?	Enter number of days
G9	How much time do you spend walking or bicycling for travel on a typical day?	Hours --<br>Minutes --
**Recreational activities**<br>The next questions exclude the work and transport activities that you have already mentioned. Now I would like to ask you about sports, fitness and recreational activities		
G10	Do you do any sports/recreational activities?	No 0<br>Yes 1<br>**If No skip to G15**
G11	Can you please list the sports/recreational activities	List the activities
G12	What is the intensity of these sports/recreational activities: vigorous-intensity - fitness or recreational (leisure) activities that cause large increases in breathing or heart rate like [running or football] for at least 10 minutes continuously or moderate-intensity – activities that cause a small increase in breathing or heart rate such as brisk walking or football for at least 10 minutes continuously.	Vigorous intensity 0<br>Moderate intensity 1
G13	In a typical week, on how many days do you do these sports/activities?	Enter number of days --
G14	How much time do you spend doing these sports/recreational activities on a typical day?	Hours --<br>Minutes --
**G. PHYSICAL ACTIVITY; Recreational Activities Continued**		
G15	Do you think physical inactivity may be associated with poor health outcomes?	No 0<br>Yes 1<br>**If No, skip to G17**
G16	Can you list the conditions or diseases that are associated with physical inactivity	Enter list ………<br>………

G17	Have you ever been told by a doctor or health worker to be physically active	No 0<br>Yes 1
**Sedentary behaviour**<br>The following question is about sitting or reclining at work, at home, getting to and from places, or with friends including time spent sitting at a desk, sitting with friends, traveling in car, bus, reading or watching television, but do not include time spent sleeping.		
G18	How much time do you usually spend sitting or reclining on a typical day?	Hours --<br>Minutes --

H. BLOOD PRESSURE/HYPERTENSION

The following questions will ask you about your history, treatment and knowledge of blood pressure/hypertension		
**Serial**	**Questions**	**Response and coding categories**
H1	Have you ever had your blood pressure measured by a doctor or other health worker?	No 0<br>Yes 1<br>**If No skip to H9**
H2	Have you ever been told by a doctor or other health worker that you have raised blood pressure or hypertension?	No 0<br>Yes 1
H3	When were you first told?	Upto 1 month 1<br>**If longer than 1 month, enter:**<br>Months --<br>Years --
H4	In the past two weeks, have you taken any drugs (medication) for raised blood pressure prescribed by a doctor or other health worker	No 0<br>Yes 1
H5	How many drugs/types of medication do you take to control blood pressure	Enter number --
H6	Do you take medication on as prescribed?	No 0<br>Yes 1<br>**If Yes skip to H8**
H7	What are the reasons for missing medication	List the reasons -------------------
H8	Where do you get your medication	Government clinic/hospital 0<br>Private hospital 1<br>Private pharmacy 2<br>Other 3

H9	Are there any dietary or lifestyle changes that you were advised by a doctor or health worker to help to control your blood pressure level?	No 0<br>Yes 1<br>**If No, skip to H13**
H10	Can you list the dietary or lifestyle changes	List of changes
H11	Do you follow the advised changes?	No 0<br>Yes 1
H12	Have you ever seen a traditional healer for raised blood pressure or hypertension?	No 0<br>Yes 1
H13	Are you currently taking any herbal or traditional remedy for your raised blood pressure?	No 0<br>Yes 1
H14	Which age group is at most risk of having hypertension?	18 – 30 years 0<br>30 – 40 years 1<br>40 – 50 years 2<br>Above 50 years 3<br>All age groups 4

J. HISTORY OF DIABETES

The following questions will ask you about your history, treatment and knowledge of type 2 diabetes		
**Serial**	**Questions**	**Response and coding categories**
J1	Have you ever had your blood sugar measured by a doctor or other health worker?	No 0<br>Yes 1
J2	Have you ever been told by a doctor or other health worker that you have raised blood sugar or diabetes?	No 0<br>Yes 1
J3	When were you first told?	Months ----<br>Years ----
J4	In the past two weeks, have you taken any drugs (medication) for diabetes prescribed by a doctor or other health worker?	No 0<br>Yes 1
J5	How many drugs/types of medication do you take to control blood pressure	Enter number
J6	Do you take medication on as prescribed?	No 0<br>Yes 1<br>**If yes skip to J8**
J7	What are the reasons for missing medication	List the reasons
J8	Where do you get your medication	Government clinic/hospital 0<br>Private hospital 1<br>Private pharmacy 2<br>Other 3
J9	Are there any dietary or lifestyle changes that you were advised by a doctor or health worker to help to control your blood pressure level?	No 0<br>Yes 1<br>**If No, skip to J12**

J10	Can you list the dietary or lifestyle changes	List of changes
J11	Do you follow the advised changes?	No 0<br>Yes 1
J12	Have you ever seen a traditional healer for diabetes or raised blood sugar?	No 0<br>Yes 1
J13	Which age group is at most risk of developing type 2 diabetes mellitus?	18 – 30 years 0<br>30 – 40 years 1<br>40 – 50 years 2<br>Above 50 years 3<br>All age groups 4

K. BODY IMAGE PERCEPTION

I shall ask you a few questions on how you feel and your perception of body image. I will ask you questions and you will choose your answer from body image pictorials from the picture I will show you. You will select one image for each question.		
**Serial**	**Questions**	**Response and coding categories**
K1	Which of these pictures do you think depicts your body?	Record choice selected
K2	If you were to change your body size, which one of the body sizes in the picture would change to?	Record choice selected
K3	Which of these pictures do you think best represents body images of people of your age and gender in Ingwavuma?	Record choice selected

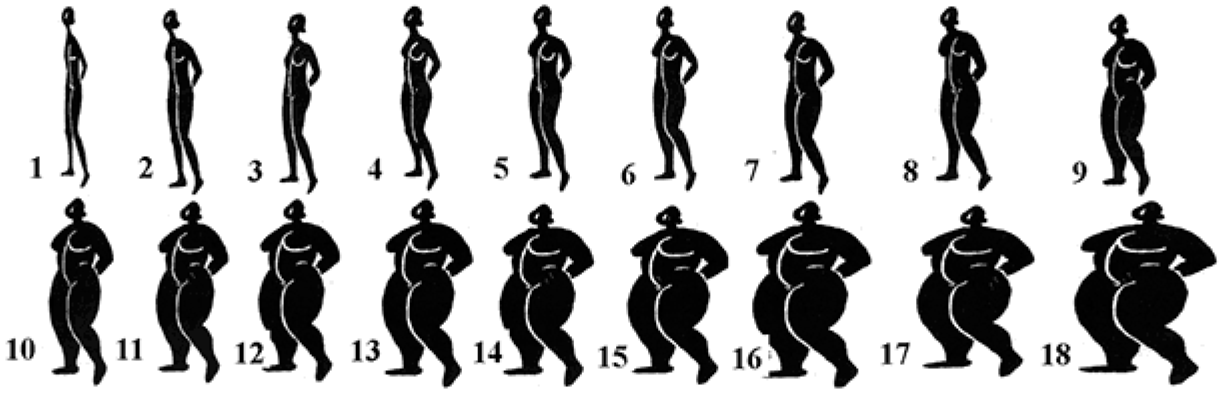

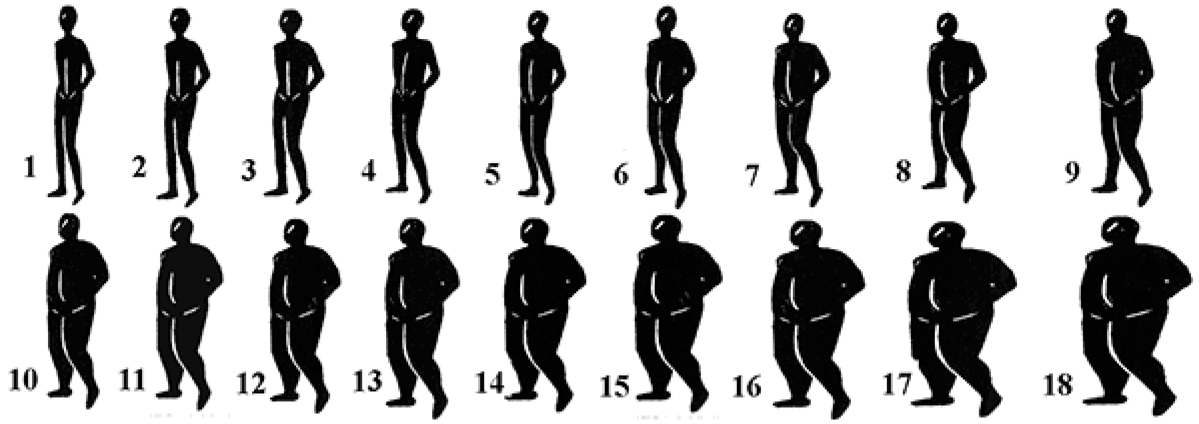

L. MEASUREMENTS

Height, weight and waist circumference

L1	Height	cm
L2	Weight	kg
L3	Waist circumference	cm

Blood pressure

		Systolic	Dystolic
L4	First Reading		
L5	Second reading		
L6	Third reading		

Blood sugar

	Haemoglobin A1c level (%)
L7	

Thank you so much for your time

Interview end time ………………………………………….(Device time)

END
