# Supplementary material for: Hypertension care cascade in the Ingwavuma rural community, uMkhanyakude District, KwaZulu-Natal province of South Africa
Source: PeerJ. 2021 Nov 11;9:e12372. doi: 10.7717/peerj.12372 (PMC8590801; doi:10.7717/peerj.12372)
Supplement: Supplemental Information 2 [file peerj-09-12372-s002.pdf]

STUDY TITLE: PREVALENCE, AWARENESS, HEALTHCARE UTILIZATION AND BURDEN OF CARDIOVASCULAR DISEASE AND ITS RISK FACTORS IN INGWAVUMA, KWAZULU-NATAL

INDIVIDUAL QUESTIONNAIRE

Igama lalocwaningayo ……………………

Isuku lokucwaninga (device date)………………..

Isikhathi sokuqala ucwaningo (device time)…………….

Isingeniso: Igama lami nginguHerbert Chikafu, ngiyinxenye yeqembu labacwaningi abavakashele indawo yaseNgwavuma, ukuze sixoxe nabantu besazise ngemibono, ulwazi lwabo kanye nokuxwayiseka mayelana nezifo, isifo senhliziyo, ukukhuluphala okwendlulele, umfutho wegazi ophezulu, kanye nesifo sikashukela esesifike esitejini sesibili.

Inhloso yalolucwaningo: ukuhlola ulwazi, imibuzo kanye nendlela yokuziphatha yabantu endaweni yaseNgwavuma mayelana nezifo ezifana noshukela owedlulele kanye nomfutho wegazi ophezulu. Lolulwazi luzosiza ukuthuthukisa ezokuphepha kulendawo kanye nokufundiswa kwabantu ngalezizifo ikakhulukazi isifo senhliziyo kanye nezimbangela zalesisifo. Ngalolulwazi kuzosizakala indawo yaseNgwavuma kanye nezinye izindawo zaseNingizimu Afrika. Isifo senhliziyo sibalelwa kwezinye eziholayo ekubeni yimbangela yokwehlukunyezwa kanye nokufa ebantwini baseNingizimu Afrika nakwamanye amazwe asakhula. Ngakhoke ukuba yingxenye yalocwaningo kungasisiza kakhulu futhi singabonga.

Imfihlo nemvume: Kunemibuzo engizoyibuza kumuntu ngamunye, imibuzo efana nayo abanye abantu bayesaba ukuyiphendula kepha ningethuki amagama enu angeke ngiwabhale phansi uma ngikubuza. Angeke futhi ngiwaqhathanise nemininingwane eningitshelwa yona. Awuphoqiwe ukuphendula imibuza ongathandi ukuyiphendula, kanti nalengxoxo ungayimisa nanoma yinini uma usozizwa sewenele. Kodwake ukwethembeka kwakho uma uphendula imibuzo kuzosisiza kube sazi ukuthi abantu bacabangani ngalezifo, esenhliziyo, esikashukela, umfutho wegazi ophezu kanye nokukhuluphala okwedlulele.

Singathokoza kakhulu ukuba nawe njengengxenye yalengxoxo. Kungathatha imizuzu engu (45) kuya ehoreni elilodwa (1hr) ukubuza imibuzo. Ungathanda ukuba yingxenye?

Yebo:	
Cha:	

………………………………………………………………………………………………

Isignesha

SECTION A: INTERVIEW DETAILS AND BACKGROUND CHARACTERISTICS

Inombolo	Imibuzo	Izimpendulo
A1	Igama lalobuzwayo	
A2	Ubulili balobuzwayo	
A3	Usuku lokuzalwa (Veza umasizi)	
A4	Iminyaka yobuzwayo (uma engenawo umazisi cela ikhadi lasemtholampilo)	
A5	Igama lendawo	
A6	Igama lesigodi	
A7	Iwadi	
A8	Inombolo yomuzi (beka Inombolo yomuzi ekhadini lamaleveva)	
A9	Inombolo yocingo	
A10	Imininingwane yokushada	Angishadile 0<br>Ngishadile 1<br>Ngihlalisene nomunye 2<br>Sehlukaniso somshado 3<br>Ngingumfelokazi 4
A11	Ingabe usuke wasifunda isikole?	Cha 0<br>Yebo 1
A12	Wagcine kuliphi ibanga kwezemfundo (khetha okukudwa)	Emabangeni aphansi 0<br>Ebangeni eliphezulu 1<br>Ekolishi 2<br>Ngaphezukwelikoshi 3
A13	Ukhona umsebenzi owenzayo	Ngingumfundi 0<br>Ngiyazisebenza 1<br>Ngisebenza ngaphansi kukahulumeni 2<br>Ngisebenzela inkapani ezimele 3<br>Angisebenzi 4<br>Ngathatha umhlalaphansi 5
A14	Bangaki abantu abasebenzayo endlini yakwenu?	Bhala Inombolo
A15	Ingabe ikhona imali yesibonelelo oyitholayo kuhulumeni?	Cha 0<br>Yebo 1

A16	Ngaphandle kwakho bangaki abantu bakini abahola igrant?	Bhala Inombolo
A17	Usuhlale isikhathi esingakanani kulendawo?	Inombolo yeminyaka |__|__| Inombolo yezinyanga |__|__|

B. IMBUZO NGEZEMPILO

Inombolo	Imibuzo	Izimpendulo
B1	Ujwayele ukulutholaphi usizo lwezempilo?	Umtholampilo mphakathi 0<br>Umtholampilo ozimele 1<br>Ikhemisi elizimele 2<br>Umthandazi 3<br>Inyanga 4
B2	Uke uhluleke ukufuna usizo lwezokuphepha uma uzizwa ugula?	Cha 0<br>Yebo 1
B3	Bala izizathu ezenza ungayi esibhendlela/ emtholampilo uma ugula	Zibale
B4	Iziphi zona izifo ezihlasele kakhulu kulendawo?	Zibale
B5	Iziphi izifo ezibulala kakhulu abantu bakulendawo?	Zibale
B6	Usuke wezwa ngesifo senhliziyo nemithambo yegazi?	Cha 0<br>Yebo 1
B7	Uyazazi izinto ezidalela ekubeni nesifo senhliziyo nemithambo yegazi?	Cha 0<br>Yebo 1<br>**If No skip to B9**
B8	Ungazibala izinto eziholela ekubeni nesifo senhliziyo?	Zibale
B9	Ingabe zikhona izindlela zokunciphisa amathuba okuba nesifo senhliziyo nemithambo yegazi?	Cha 0<br>Yebo 1
B10	Ungazibala izindlela zokwehlisa amathuba okuphathwa yisifo senhliziyo nemithambo yegazi?	Zibale
B11	Abantu abaneminyaka emingaki abasengozini yokuphathwa yisifo senhliziyo nemithambo yegazi?	18 – 30 iminyaka 1<br>30 – 40 iminyaka 2<br>40 – 50 iminyaka 3<br>Ngaphezu kwengu 50 4<br>Bonke abantu 5

C. UGWAYI

Sengizobuza imibuzo mayelane Chakubhenywa kukagwayi		
**Inombolo**	**Imibuzo**	**Izimpendulo**
C1	Ingabe lukhona uhlobo lukagwayi olubhemayo (imboza, inqawe)?	Cha 0<br>Yebo 1<br>**Uma kungucha welela c8**
C2	Ingabe ubhema nsuku zonke?	Cha 0<br>Yebo 1
C3	Waqala uneminyaka emingaki ukubhema?	Bhala iminyaka<br>Angazi
C4	Mawungalinganisa isibalo sezinhlobo zogwayi ozibhema ngosuku zingaki?	Bhala inombolo yo:<br>Gwayi othengiwe<br>Gwayi wekuzigocela<br>Gwayi wenqawe<br>Okunye
C5	Ezinyangeni ezilinganiselwe onyakeni ezendlule usuke wazama ukuyeka ugwayi?	Cha 0<br>Yebo 1
C6	Uke wavakashela udokotela noma umsizi wezempilo ezinyangeni ezilingene unyaka ezindlulile?	Cha 0<br>Yebo 1
C7	Ngenkathi uvakashele umtholampilo bakwexwayisa yini ukuba uyeke ugwayi?	Cha 0<br>Yebo 1
C8	Ngenkathi endlule wake wawubhema ugwayi?	Cha 0<br>Yebo 1<br>**Uma kungucha welela C12**
C9	Enkathini endlule ububhema nsuku zonke?	Cha 0<br>Yebo 1
C10	Wayeka unemnyaka emingaki ukubhema?	Bhala iminyaka<br>Angazi 9
C11	Usuqede isikhathi esingakanani wayeka ukubhema?	Iminyaka<br>Izinyanga<br>Amaviki<br>Angazi 9
C12	Ingabe njengamanje uyayisebenzisa into esagwayi kepha enganantuthu (efana nesinemfu / ugwayi ohlafunwayo)?	Cha 0<br>Yebo 1
C13	Ucabanga ukuthi ukubhema ugwayi kungasidala isifo esingelapheki noma okungakhulumeki ngaso?	Cha 0<br>Yebo 1<br>**Uma kungucha welela D1**
C14	Yiziphi izifo ezingelapheki ezidalwa ukubhema ugwayi?	Zibale

D. UKUPHUZA KWAMANZI AMPONJWANA

Ngizokubuza imibuzo ephatheleni nokuphuzwa kwamanzi amponjwana		
**Inombolo**	**Imibuzo**	**Izimpendulo**
D1	Uke waphuza utshwala obufana nobhiya newayini?	Cha 0<br>Yebo 1<br>**Uma kungucha welela ku E1**
D2	Waphuza uhlobo olunjani lotshwala?	Ubhiya 0<br>Yiwayini 1<br>ugologo 2<br>Umqombothi 3<br>Nalezinye 4
D3	Usuke waphuza utshwala ezinyangeni ezingange shumi nambili yezedlulile?	Cha 0<br>Yebo 1<br>**Uma kungucha welela ku E1**
D4	Uke wayeke ukuphuza ngezizathu eziphathelene nokugula noma ngenxa yezaluleko ezibuya kudokotela noma umhlengikazi?	Cha 0<br>Yebo 1
D5	Ezinyangeni ezingangelishumi nambili, uphuze kangaki iziphuzo ezinotshwala?	Nsuku zonke 0<br>5-6 izinsuku ngesonto 1<br>3-4 izinsuko ngesonto 2<br>1-2 izinsuko ngesonto 3<br>1-3 izinsuko ngenyanga 4
D6	Usuke waphuza enyangeni endlule?	Cha 0<br>Yebo 1<br>**Uma kungucha welela ku D12**
D7	Kulenyanga endlule ubuphuza kangakanani?	Bhala inombolo<br>Angazi 9
D8	Utshwala ngabunye ezihlobeni ozijwayele, ubuphuza kangaki kulenyanga eyedlule?	Bhala inombolo<br>Angazi 9
D9	Ithini inombolo enkulu kunazo zonke yotshwala oke wabuphuza emcimbini uwodwa kulenyanga endlule?	Bhala inombolo<br>Angazi 9
D10	Kulenyanga eyedlule uke waphuza utshwala obulinganiselwe esithupheni noma ngaphezulu ngosuku ololadwa kangaki?	Bhala inombolo<br>Angazi 9

D11	Ezinsukwini eziyisikhombisa ezendlule uke waphuza utshwala obungakanani ngezinhlobo zabo?	Bhala inombolo ngosuku<br><br>Msumbuluko 0<br>Lwesibili 1<br>Lwesithathu 2<br>Lwesine 3<br>Lesihlanu 4<br>Umgqibelo 5<br>Isonto 6
D12	Onyakeni owedlule uzame wehluleka kangaki ukuyeka utshwala?	Angazi 0<br>Kaningi 1<br>Kamubalwa 2
D13	Enyakeni owedlule ubudinge kangaki utshwala bokuqeda ibhabhalaza emva kokulalala uphuze kakhulu	Akaze 0<br>Cishe kube nsuku zonke 1<br>Njalo ngenyanga 2<br>kungaphansi kwenyanga<br>Njalo ngeviki
D14	Usuke wayeka ukuphuza?	Cha 0<br>Yebo 1<br>**Uma kunguCha welela ku E1**
D15	Yini eyenza uyeke ukuphuza?	Zibale

E. UKUDLA KWEMPILO

Sengizobuza imibuzo ephathelene nezakhamzimba (Ukudla)		
**Inombolo**	**Imibuzo**	**Izimpendulo**
E1	Uyazidla izithelo	Cha 0<br>Yebo<br>uma kunguCha welela ku **F1**
E2	Evikini uzidla kangaki izithelo?	Bhala inombolo<br>Angazi 9
E3	Ngosuku ujwayele ukudla izithelo ezinganani?	Bhala inombolo<br>Angazi 9
E4	Uyayidla imifino?	Cha 0<br>Yebo 1<br>**Uma kunguCha welela ku F1**
E5	Evikini, uzidla kangaki izitshalo/amaveji?	Bhala inombolo<br>Angazi 9
E6	Udla inani elingakanani lamaveji ngosuku?	Kancane 0<br>Kakhulu 1<br>Okulingene 2

F. USAWOTI WOKUDLA

Ngale mibuzo elandelayo, sithanda ukuzwa kabanzi mayelana nosawoti/notswayi ekhudleni kwako. I-Dietary salt ikhuluma ngalo lonke uhlobo lukasawoti, usawoti omahadladladla, usawoti o-iodized, amaqhezu estock, nosawoti owupowda, kanjalo namasosi anosawoti. Lemibuzo ingokufaka usawoti ekudleni ngaphambi kokudla, nasekutheni ukudla ukwenza kanjano kwakho, ekudleni okukhiqiziwe okunosawoti omningi njengama chips, amazambane athosiwe, kanjalonokulawula indlela ofaka ngayo usawoti. Ngicela uphendule le mibuzo ngisho noma ngabe wena uwudla ndendlela usawoti.		
**Inombolo**	**Imibuzo**	**Izimpendulo**
F1	Uwufaka kangaki usawoti noma isombo elinosawoti ngaphimbi kokudla noma uma udla?	Angifaki 0<br>Ngalesinye isikhathi 1<br>Sonke sikhathi 2
F2	Uzisebenzisa kangaki izonongo ezinosawoti, ezifana no-Aromat, iknorrox, uma upheka?	Angifaki 0<br>Ngalesinye isikhathi<br>1<br>Sonke sikhathi<br>2
F3	Ukudla kangaki ukudla okugcinwe ngosawoti ukuze kungaboli ukudla okufana nofishi wethini, oshizi, ubhekeni, kanye nokudla kwase zitolo ezidayisa ukudla okuphekiwe	Angifaki 0<br>Ngalesinye isikhathi 1<br>Sonke sikhathi 2
F4	Ufaka izinandisi ezinosawoti ezingakanani ekudleni?	Ezincane kakhulu 0<br>Ezincane 1<br>Isikalo esilingene 2<br>Eziningi 3<br>Eziningi kakhulu 4
F5	Ingabe kubaluleke kanganani ukwehlisa usawoti?	Akubaluleki neze 0<br>Khona kubalulekile 1<br>Kubaluleke kakhulu 2<br>Angazi 9
F6	Ucabanga ukuthi ukudla okunosawoti kakhulu kungakubangela izifo?	Cha 0<br>Yebo 1<br>Angazi 9

		**Uma kunguCha welela ku F8**
F7	Uma ukhethe uyebo, yiziphi lezifo ezingakuphatha?	Zibale
F8	Uyazikhethela ukuthenga ukudla okugcinwe ngasowoti ukuze kungaboli?	Cha 0<br>Yebo 1
F9	Uyalibheka izinga lasawoti ekudleni uma ukuthenga?	Cha 0<br>Yebo 1
F10	Uyakuthenga ukudla okunosawoti omuncane?	Cha 0<br>Yebo 1
F11	Uyazisebenzisa izinongo ezinganasawoti uma upheka?	Cha 0<br>Yebo 1
F12	Uyakugwema ukudla okuphekwe ezitolo?	Cha 0<br>Yebo 1
F13	Kukhona okwenzayo ukuze ulawule izingo losawoti emzimbeni?	Cha 0<br>Yebo 1
F14	Iziphi lezozinto ozenzayo?	Zibale

G. UMSEBENZI WOMZIMBA

Okulandelayo ngizobuza mayelana ngemisebenzi oyenza ngomzimba wakho. Lemisebenzi ifana nokuzihambela ebaleni, ukugijima(ukuzivocavoca) kanye nokwenza umsebenzi wansu zonke ekhaya efana nokupheka, nokushanela, nokuwasha, kanye nokunye okuninngi okwenza ngomzimba wakho okukukwenza uhefuzela noma inhliziyo ishayele phezulu.		
**Inombolo**	**Imibuzo**	**Izimpendulo**
G1	Ingabe imisebenzi yakho ibala nokugubha imigodi, ukuphakamisa izinto ezisindayo kanye nokusebenza ezinkontileleni imizuzu edlule ulelishumu, okuze kwenze inhliziyo ishaye ngamandla kanye nokuphefumulela phezulu.	Cha 0<br>Yebo 1<br>**Uma kunguCha welela ku G5**
G2	Lemisebenzi uyenza kangaki evikini?	Bala
G3	Uchitha isikhathi esinganani wenza lemisebenzi?	Amahora --<br>Imizuzu --
G4	Ingabe imisebenzi oyenzayo ayibali kusebenza okunganyakazisa inhliziyo nokuhefuzela okwedlula imizuzu elishumi?	Cha 0<br>Yebo 1
G5	Evikini uyenza kangakanana imisebenzi engakunyakazisa kakhulu?	Bhala izinsuku
G6	Uchitha isikhthi esingakanani wenza imisebenzi engakhathazi emsebenzini?	Amahora --<br>Imizuzu --

**Ukuya nokubuya ezindaweni**<br>Lemibuzo elandelayo imayelana nokuhamba nokubuya ezindaweni isibonelo, ukuyothenga ukuya emsebenzini njalonjalo		
G7	Uyahamba noma uyagibela ibhayisikili ukuze uye ezindaweni isikhathi esidlule emizuzwini elishumi?	Cha 0<br>Yebo 1
G8	Ujwayela ukuhamba kangaki noma kugibela kangaki ibhayisikili evikini?	Bhala inombolo
G9	Uthatha isikhathi esinganani uhamba ngeziyawo noma ngebhayisikili osukwini?	Amahora --<br>Imizuzu --
**Ukuzivocavoca**<br>Lemibuzo elandayo imayelana nezokuzivocavoca kwezemidlalo		
G10	Uyayidlala imidlalo?	Cha 0<br>Yebo 1<br>**Uma kunguCha welela ku G15**
G11	Bala imidlalo oyenzayo?	Zibale
G12	Ingabe lomdlalo owenzayo uthatha amandla amangakanani	Amandla amancane 0<br>Amandla amaningi 1
G13	Lemidlalo uyenza isikhathi esinganani evikini?	Faka inombolo yezinsuku --
G14	Osukwini uthatha isikhathi esinganani udlala lemidlalo?	Aamahora --<br>Imizuzu --
G15	Ukucabanga ukuthi ukungazivocavoci kungaholela ekuphathweni yizifo ezithize?	Cha 0<br>Yebo 1<br>**Uma kungucha welela ku H1**
G16	Ungazibala izifo ezithize ezingadalwa ukungazivocavoci?	Zibale
G17	Usuke waxwayiswa udokotela/ umhlengikazi ukuba uzijwayeze ukuzivocavoca?	Cha 0<br>Yebo 1
**UHLALA PHANSI**<br>Lemibuzo elandelayo imayelana nesikhathi esichithwa uhleli, kungaba yisekhaya ubuka umabona kude nebangani, ingaba kusebhansini/ imoto uyahamba, kanye nokuba sesikoleni uhleli ufunda, odwa loku akuthintani nesikhatsi sekulala		
G18	Singanani isikhathi osicithayo uhleli phansi osukwini?	Amahora --<br>Imizuzu --

H. UMFUTHO WEGAZI

Imibuzo elandelayo imayelana nolwazi kanye nokwelashwa komfutho wegazi ophezulu		
**Inombolo**	**Imibuzo**	**Izimpendulo**
H1	Usuke wahlolwa ngudokotela ekuhloleka umfutho wegazi?	Cha 0<br>Yebo 1<br>**Uma kunguCha welela ku H9**
H2	Usuke watshelwa udokotela noma abahlengikazi ukuthi unomfutho wegazi ophezulu ngokwedlulele	Cha 0<br>Yebo 1
H3	Watshelwa nini?	Enyangeni endlule 1<br><br>**Uma kungaphezu kwenyanga:**<br>Bhala izinyanga<br>iminyaka
H4	Emavikini amabili edlule esuke wathatha imithi yokwe lapha isifo somfutho wegazi ophezi ngemiyalelo yadokotela?	Cha 0<br>Yebo 1
H5	Wadla izihlobo ezingaki zemithi ukuze ulawule lokugula	Inombolo --
H6	Imithi uyisebenzisa njengemiyalelo?	Cha 0<br>Yebo 1<br>**Uma kungu yebo welela ku H6**
H7	yiziphi izizathu zokungayidli imithi?	Zibale
H8	Uyitholaphi imithi	Esibhedlela sikahulumeni<br>Esibhedlela esizimele<br>Ikhemisi<br>Okunye
H9	Igabe usuke waqhushiswa ngudokotela ukuba usebenzise indlela ethize yokudla ukuze ulawule umfutho wegazi ophezulu?	Cha 0<br>Yebo 1<br>**Uma kungu cha welela kuH13**
H10	Ungazisho lezindlela/ zinhlobo zokudla	Zibale

H11	Uyayilandela imiyalelo yadokotela?	Cha 0<br>Yebo 1
H12	Usake wabonana nenyanga mayelana nesifo semfutho wegazi ophezulu?	Cha 0<br>Yebo 1
H13	Ingabe zikhona izimbiza zesintu oziphuzayo mayelana nokulapha lesifo?	Cha 0<br>Yebo 1
H14	Abantu beminyanga emingakanani abase ungozini bokuba nesifo somfutho wegazi ophezulu?	18 – 30 iminyaka 0<br>30 – 40 iminyaka 1<br>40 – 50 iminyaka 2<br>Abangaphezulu kwengu 50 3<br>Bonke abantu 4

J. UMLANDO WESIFO SIKASHUKELA

Lemibuzo elandelayo iphathelene nomlando wakho noshukela		
**Inombolo**	**Imibuzo**	**Izimpendulo**
J1	Usuke wahlolwa ngudokotela akuhlolela ushukela?	Cha 0<br>Yebo 1
J2	Basake basho abahlengikazi noma odokotela ukuthi unoshukela ophezulu	Cha 0<br>Yebo 1
J3	Bakwazisa nini?	Izinyanga<br>Iminyaka
J4	Emavikini amabili edlule ikhona imithi yokulapha ushukela okuyisebenzisa ngemiyalelo yadokotela	Cha 0<br>Yebo 1
J5	Uthatha izinhlobo ezingaki zemithi ukuze ulawule ushukela?	Bhala inombolo
J6	Imithi uyidla ngedlela oyalelwe ngayo?	Cha 0<br>Yebo 1<br>**Uma kungu yebo welela kuJ8**
J7	Yiziphi izizathu zokungadli imithi ngemiyalelo?	Zibale
J8	Uyitholaphi imithi?	Emtholampilo kahlumeni 0<br>Emthalampilo Ozimele 1<br>-Ekhemisi elizimele 2<br>Okunye 3
J9	Ingabe wexwayiswa ngokushintsha indlela udla ngayo ngodokotela ngenxa yesifo sikashukela?	Cha 0<br>Yebo 1<br>**Uma kunguCha welela kuJ12**
J10	Ungazibala lezindlela zokudla	Zibale
J11	Uyailandela imiyalelo yadokotela	Cha 0<br>Yebo 1
J12	Usuke waya enyangeni uyelashwa ushukela?	Cha 0<br>Yebo 1

J13	Abantu beminyanga emingakanani abase ungozini bokuba nesifo soshukela?	18 – 30 iminyaka 0<br>30 – 40 iminyaka 1<br>40 – 50 iminyaka 2<br>Abangaphezulu kwengu 50 3<br>Bonke abantu 4

K. UMBONO NGOMZIMBA

Ngizokubuza imibuzo ephatheleni nemizwa kanye nembono wakho mayelana nendlela imizimba ime ngayo. Uzongiphendula ngokubhala inombolo yesithombe lesihambisana nempendulo yakho.		
**Inombolo**	**Imibuzo**	**Izimpendulo**
K1	Yisiphi isithombe ocabanga ukuthi siveza noma sifana nomzimba wakho	Bhala isithombe osikhethile
K2	Uma ungashitsha isisindo somzimba wakho, ungashitshela kusiphi isithombe?	Bhala inombolo yesithombe losikhethile
K3	Yiziphi izithombe ocabanga ukuthi ziveza imizimba yabantu baseNgwavuma?	Bhala inombolo yesithombe osikhethile

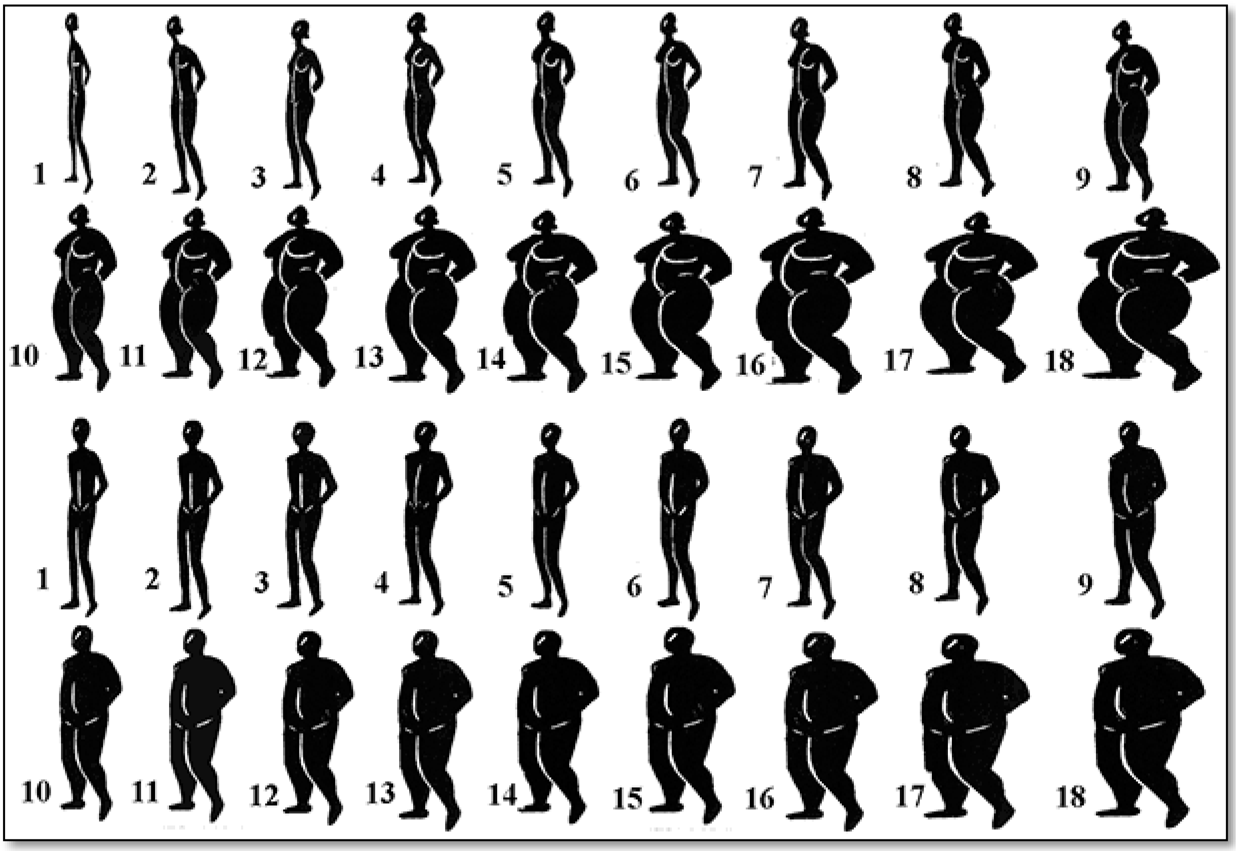

L. ISIKALO

UBUDE, ISISINDO, UKHALO

L1	Ubude	cm
L2	Isisindo	kg
L3	Ukhalo	cm

Umfutho wegazi

		I-Systolic	i-Dystolic
L4	Ukufunda okuqala		
L5	Ukufunda kwesibili		

L6	Ukufunda kwesithathu		

Shukela wegazi

	i-hemoglobin Alc level (%)
L7	

Ngiyabonga kakhulu ngesikhathi sakho

Isikhathi sokuphela kocwaningo …………………………………………….(Device time)
