# Supplementary material for: Hypertension care cascade in the Ingwavuma rural community, uMkhanyakude District, KwaZulu-Natal province of South Africa
Source: PeerJ. 2021 Nov 11;9:e12372. doi: 10.7717/peerj.12372 (PMC8590801; doi:10.7717/peerj.12372)
Supplement: Supplemental Information 4 [file peerj-09-12372-s004.docx]

VARIABLES

This data with 131 hypertensive participants was extracted from the master file that 391 complete entries from a sample of 400 participants. The study being on the hypertension care cascade comprising screening, diagnosis/awareness, treatment and control, hence the extraction on relevant data. Extraction also helped to reduce the file size for ease of uploading.

In the data set, variables used in the analysis are prefixed with ‘VAR’ and are provided in the legend below.

| **VARIABLE** | **DESCRITION** | **CODES and CATEGORIES** |
| --- | --- | --- |
| VAR_AGE | Participant age | - 0 - 18-39 - 1 - 40-59 - 2 - 60+ |
| VAR_GENDER | Participant gender | - 0 - Female - 1 - Male |
| VAR_PA | Participant weekly physical activity level | - 0 - Insufficient/inactive - 1 - Sufficiently active - 2 - Highly active |
| VAR_EDU | Participant formal education level | 0 - No formal education  - 1 - Primary education - 2 - Post |
| VAR_EMPLOY | Participant employment status | - 0 - Not employed - 1 - Employed (self-employed and formally employed) |
| VAR_MARITALSTATUS | Participant marital status | - 0 - Not in union - 1 - Married - 2 - Cohabiting |
| VAR_ DIABETES | Participant diabetes mellitus status | - 0 - No - 1 - Yes |
| VAR_BMI | Participant body mass index | - 0 - Up to normal weigh - 1 - Overweight - 2 - Obese |
| VAR_ALC_CONSUMPTION | Current alcohol consumption status defined by alcohol consumption in 30 days preceding the interview | - 0 - No - 1 - Yes |
| VAR_SMOKE | Current tobacco smoking status defined by tobacco smoking in 30 days preceding the interview | - 0 - No - 1 - Yes |
| VAR_SCREEN | Whether participant had ever had blood pressure checked by a health profession at any time | - 0 - No - 1 - Yes |
| VAR_DIAG | For participants who had been screened by a health worker, whether participant was told of their hypertensive status | - 0 - No - 1 - Yes |
| VAR_TRT | Current pharmacological treatment for hypertension - participants who had been diagnosed with hypertension, and were informed of their hypertensive status. | - 0 - No - 1 - Yes |
| VAR_CONTROL | For diagnosed participants under pharmacological treatment for hypertension, whether they have controlled hypertension | - 0 - No - 1 - Yes |
